# Association between menstrual disorders and anti-Müllerian hormone with COVID-19: A prospective cohort study

**DOI:** 10.18502/ijrm.v22i10.17669

**Published:** 2024-12-02

**Authors:** Ziba Haghipour, Shahideh Jahanian Sadatmahalleh, Fatemeh Razavinia, Malihe Nasiri

**Affiliations:** ^1^Department of Reproductive Health and Midwifery, Faculty of Medical Sciences, Tarbiat Modares University, Tehran, Iran.; ^2^Midwifery Department, Reproductive Health Promotion Research Center, Nursing and Midwifery School, Ahvaz Jundishapur University of Medical Sciences, Ahvaz, Iran.; ^3^Department of Basic Sciences, Faculty of Nursing and Midwifery, Shahid Beheshti University of Medical Sciences, Tehran, Iran.

**Keywords:** COVID-19, AMH, TSH, TPO, Prolactin, Menstrual disorders.

## Abstract

**Background:**

Coronavirus 2019 (COVID-19) has affected almost all communities throughout the world. It affects different systems in the body, which causes irreparable consequences.

**Objective:**

This study aimed to investigate the effect of the COVID-19 virus on menstrual disorders, anti-Mullerian hormone (AMH), thyroid peroxidase (TPO), thyroid stimulating hormone (TSH), and prolactin levels in women with COVID-19 disease.

**Materials and Methods:**

This prospective cohort study was conducted on 201 women (101 healthy, and 100 women with COVID-19) in Nomadic hospital, Khorramabad, Iran from February-October 2021. After recovery from COVID-19, participants were interviewed about their menstrual cycle in their 1 ${\;}^{\text{st}}$ and 4 ${\;}^{\text{th}}$ months. Blood samples were collected during 1 ${\;}^{\text{st}}$ and 4 ${\;}^{\text{th}}$ months, and AMH, TSH, TPO, and prolactin levels were assessed.

**Results:**

No significant differences were observed in the 1 ${\;}^{\text{st}}$ and 4 ${\;}^{\text{th}}$ months regarding menstrual disorders, TPO, and prolactin levels in the patient group (p > 0.05). The mean level of AMH in the healthy group was higher than the patient group in both times (p < 0.001). A relationship between TSH and COVID-19 was observed in the 1 ${\;}^{\text{st}}$ month (p < 0.001); however, no significant relationship was observed in the 4 ${\;}^{\text{th}}$ month, in this regard.

**Conclusion:**

Endocrine dysfunction in the form of low AMH and high TSH were common among COVID-19 patients. Due to the importance of menstrual and hormonal disorders, especially AMH and TSH, and their association with COVID-19, health policymakers should find appropriate solutions to reduce complications.

## 1. Introduction

The coronavirus 2019 (COVID-19) pandemic mainly causes infections of the nose, throat, bronchi, and lungs, leading to cold symptoms and severe pneumonia (1). It may also involve other parts of the body such as neural, vascular, gastrointestinal, and reproductive systems, and thus, cause coagulation and gynecological disorders (2). Furthermore, there is the possibility of the involvement of female organs, including the uterus, ovaries, fallopian tubes, etc. (3). Organs with high angiotensin-converting enzyme 2 (ACE2) expressions might be attacked by COVID-19 (4). A study reported that ACE2 is expressed in ovarian granulosa cells, thus, reproductive endocrine function may be injured in this infection (5, 6). Also, menopausal women's estrogen and anti-Mullerian hormone (AMH) levels were inversely linked with the severity of COVID-19 (7). ACE2 is expressed in the thyroid gland (8), which probably affects hormonal disorders and makes changes in the hypothalamic-pituitary-thyroid axis such that COVID-19 virus has been detected in the pituitary gland post mortem (9). It is also known to damage the follicular and parafollicular cells of the thyroid at post mortem and probably causing autoimmune disease, which causes changes in the thyroid hormone levels and thyroid peroxidase (TPO) (1).

Moreover, the COVID-19 pandemic period led to an increase in the amount of stress, anxiety, and depression, affecting women more than men (10). Studies have shown that external factors such as stress and anxiety affect the hypothalamic-pituitary-ovarian axis, causing changes in the pulsating secretion of hormones such as follicle-stimulating hormone, luteinizing hormone, and menstrual disorders (11, 12).

So far, the COVID-19 virus has infected large populations throughout the world. Because the disease is unknown, it is important to identify its effects and complications. On the other hand, several studies have shown the effect of COVID-19 on the reproductive system and fertility (13–15).

Due to the high number of COVID-19 patients and the economic, physical, and psychological burden of reproductive and fertility diseases, we decided to investigate the effect of the COVID-19 virus on menstrual disorders and AMH, TPO, thyroid stimulating hormone (TSH) and prolactin levels in women with the COVID-19 disease. Therefore, the current study aimed to assess the menstrual disorder and the AMH, TSH, TPO, and prolactin levels in female patients with COVID-19.

## 2. Materials and Methods 

### Study design 

**Figure 1:**
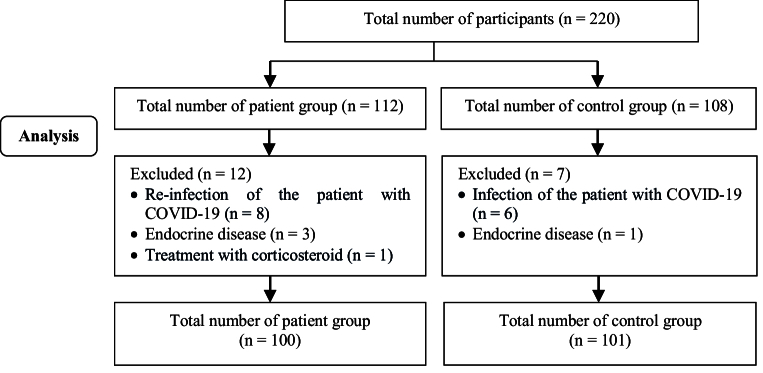
Flowchart of the present prospective cohort study.

This prospective cohort study was conducted on 201 women (101 healthy [negative polymerase chain reaction (PCR)] and 100 women with COVID-19). All participants were discharged from the hospital after the improvement in clinical and para-clinical symptoms (erythrocyte sedimentation rate, C-reactive protein) and the PCR test was not performed again to become negative. In fact, the participants were confirmed cases of COVID-19 in Nomadic hospital, Khorramabad, Iran from February-October 2021 (Figure 1).

### Sample size

The sample size was determined by a pilot study with 95% confidence interval and 80% power test as follows:

α = 0.05, Z_α/2_ = 1.96, Z_β_ = 1.28, β = 0.10, 1-β = 0.90

n ≥ 2 (Z_α/2_ + Z_β_)^2^ σ^2^/(μ_1_ - μ_2_)^2^

The size of the observed effect:

(μ_1_ - μ_2_)/σ = 0.46

### Inclusion criteria

Participants aged between 18 and 40 yr with COVID-19, admitted to the ward, lack of vaccination, and had Iranian nationality were included in the study.

### Exclusion criteria

Participants with a lack of desire to participate in the study, pregnancy at the time of study, being in a high-risk group, including treatment with corticosteroids ( > 12.5 mg/wk of prednisolone), having a history of malignancy and chemotherapy, organ transplants, having endocrine diseases such as diabetes, hyperthyroidism, and hyper prolactinemia (according to the medical records), suffering from HIV and Hepatitis B or C, body mass index (BMI) > 40, re-infection of the patient with COVID-19 during the study, having a history of drug and alcohol addiction, and having menstrual disorders or menopausal symptoms and complaints before COVID-19, were excluded from the study.

### Study design

The participants having the inclusion criteria were trained about how to fill out checklists and charts of menstrual disorders. All the participants were people who needed hospitalization due to the severity of the disease. All patients, that is, the case group, were admitted according to clinical symptoms and a positive PCR test. In other words, only one PCR test was performed for the patient group. Also, the PCR test was performed once at the beginning of the study for people who did not have clinical symptoms, and these people were included in the control group. After recovery from COVID-19, interviews were conducted about their menstrual cycle in the 1 ${\;}^{\text{st}}$ and 4 ${\;}^{\text{th}}$ months. The healthy group interview was conducted during the collection of the patient group. Demographic fertility questionnaires were completed (at the beginning of the study), and blood pressure and BMI were assessed using standard instruments. To evaluate menorrhagia, sanitary pads were given to the women to be used in 4 ${\;}^{\text{th}}$ month's cycle. The Pictorial Blood Loss Assessment Chart (PBLAC) was utilized to evaluate menstrual blood loss (16). This chart records the daily amount of menstrual bleeding by noting the number of clots and the extent of staining on each pad or tampon. All participants completed their charts for one menstrual cycle, using the same sanitary products. Due to the need to use the same pads in both groups, the amount of menorrhagia was measured only in the 4 ${\;}^{\text{th}}$ month between the 2 groups (17).

Then, the changes in bleeding were charted. Both groups of participants were examined for menstrual disorders for 4 months (Figure 1).

Definitions and terminology for menstrual pattern:

▪ A normal menstrual cycle is characterized by an interval of 21 to 35 days and menstrual duration of 7 days or less (14).

▪ The menstrual cycle length is defined as the number of days from the onset of menstruation to the beginning of the next period (14).

▪ Menstrual irregularities occur when the interval between 2 periods is either less than 21 days or more than 35 days (18).

▪> Menorrhagia is defined as a PBLAC score of 100 or higher (19).

### Laboratory measurements

Blood samples (5 ml) were taken and centrifuged (4000 rpm for 10 min). Then the serum was separated and frozen (-70 C) to evaluate the changes in AMH (ng/ml), TSH (Ulu/ml), TPO (IU/ml), and prolactin (ng/ml) levels by enzyme-linked immunosorbent assay. 4 months later, 5 cc of blood was taken from both groups again.

### Ethical Considerations

The research was approved by the Ethics Committee of Tarbiat Modares University of Medical Sciences, Tehran, Iran (Code: IR.MODARES.REC.1399.162). The study was conducted according to the 1964 Helsinki declaration and the ethical standards of the Regional Research Committee. After the study was explained, the informed consent form was completed. Their participation was voluntary, confidential and anonymous and they had the right to withdraw.

### Statistical Analysis

Data processing and statistical analysis were conducted using the Statistical Package for Social Science (SPSS, ver. 23, SPSS Inc., and Chicago, IL, USA). The normality was assessed by using the Kolmogorov-Smirnov test. The obtained data are presented as mean ± SD and number (percentage). The relationship between the study variables was investigated using *t* test, Mann-Whitney U test, Chi-square test, Fisher's exact test, multivariate linear regression, and Wilcoxon Sum-Rank tests, and p < 0.05 was considered significant.

## 3. Results

The 2 groups were matched according to their demographic characteristics as shown in table I. The average age of the participants was 34.54 ± 7.3 yr. More than half of the participants were married (75%), had postgraduate education and bachelor's degree (55.5%), and were housewives (62%). Table I shows that a significant difference was observed between the 2 groups in the distribution of varying levels of the participants' menstrual status (p < 0.001).

In addition, in table II, menstrual duration in the 1 ${\;}^{\text{st}}$ or 4 ${\;}^{\text{th}}$ month showed no significant difference between the patient and healthy groups and between the 2 times (p > 0.05).

No significant difference was observed in the menstrual cycle length in the 1 ${\;}^{\text{st}}$ and 4 ${\;}^{\text{th}}$ months in both groups (p > 0.05), and changes in the cycle length were not significant in the patient group between the 2 times. The mean score of PBLAC is not statistically significant between the patient and healthy groups in the 4 ${\;}^{\text{th}}$ month. The mean level of AMH (ng/ml) in the healthy group was significantly higher than in the patient group in the 1 ${\;}^{\text{st}}$ and 4 ${\;}^{\text{th}}$ months (p < 0.001). Also, a significant difference was observed in the mean AMH level of the patient group in the 1 ${\;}^{\text{st}}$ and 4 ${\;}^{\text{th}}$ months (p = 0.02) and the mean level of AMH in the 1 ${\;}^{\text{st}}$ month was higher than in the 4 ${\;}^{\text{th}}$ month; however, the healthy group's mean AMH level shows no significant difference in the 1 ${\;}^{\text{st}}$ and 4 ${\;}^{\text{th}}$ months (p = 0.34).

The mean level of TSH (Ulu/ml) in the healthy and patient groups for the 1 ${\;}^{\text{st}}$ month, showed a statistically significant difference (p < 0.001). Also, a significant difference was observed in the mean TSH level of the healthy participants in the 1 ${\;}^{\text{st}}$ month (p < 0.001). The results of the TSH level comparison in the 4 ${\;}^{\text{th}}$ month were not significant between the patient and healthy groups (p = 0.13) the healthy group's mean TSH level showed no significant difference in the 1 ${\;}^{\text{st}}$ and 4 ${\;}^{\text{th}}$ months (p = 0.08). The mean TPO level (IU/ml) in the patient group in the 1 ${\;}^{\text{st}}$ month was significantly higher than in the healthy group.

Multiple regression models showed that after removing the effect of confounding variables, statistically significant differences were observed between the 2 groups in menstrual length (p = 0.02) and AMH (p = 0.002). However, no statistically significant difference was observed in menstrual duration, TSH, TPO, or prolactin (Table III).

**Table 1:** Characteristics of the participants

**Variables**		**Patient (n = 100)**	**Healthy (n = 101)**	**Median**	**P-value**
Age (yr)*		35.37 ± 0.72	33.71 ± 6.97	36	0.10
Age of first pregnancy (wk)**		25.12 ± 4.65	25.25 ± 2.58	25	0.26
Menarche age (yr)**		13.29 ± 0.80	13.19 ± 0.74	13	0.39
Gravid**		1.78 ± 1.57	1.36 ± 1.21	1.5	0.43
Para**		1.40 ± 1.14	1.20 ± 1.01	1	0.21
Abortion**^a^		0.25 ± 0.43	0.15 ± 0.35	0	0.054
Live child**^b^		1 ± 1.11	1.12 ± 0.98	1	0.30
BMI (Kg/m^2^)*		25.64 ± 3.93	25.44 ± 3.05	26.17	0.10
Menstrual duration**		6.31 ± 1.13	6.66 ± 0.65	1	0.01
Women's income (Tomans)** ${\;}^{\text{c}}$		2.11 ± 2.97	1.94 ± 2.85	0	0.72
Contraceptive duration (month)**		61.79 ± 42.97	60.80 ± 42.78	48	0.80
Education***					
	**Diploma and less than a diploma**	45 (45)	35 (34, 4)		
	**Master's and Bachelor's degree**	49 (49)	62 (61.8)		
	**Master's and Doctoral degrees**	6 (6)	(3.8)		0.33
Marital status***					
	**Single**	24 (24)	25 (24.75)			
	**Married**	76 (76)	76 (75.25)		0.86
Job****					
	**Housewife**	58 (58)	67 (66)		
	**Freelance**	3 (3)	5 (5)		
	**Employed**	39 (39)	29 (29)		0.26
City of residence****					
	**Khorramabad**	94 (94)	98 (97.03)			
	**Other cities**	6 (6)	3 (2.97)		0.49
Type of housing***					
	**Rental**	13 (13)	16 (15.84)		
	**Private**	87 (87)	85 (84.16)		0.54
History of pregnancy leading to childbirth***					
	**Yes**	69 (69)	32 (31.68)		
	**No**	31 (31)	69 (68.32)		0.87
Type of delivery***					
	**NVD**	43 (62.3)	40 (57.98)		
	**CS**	26 (37.7)	29 (42.02)		0.55
Contraceptive methods***					
	**OCP**	6 (7.9)	14 (18.42)		
	**IUD**	8 (10.5)	8 (10.52)		
	**Others**	62 (81.6)	54 (71.06)		0.13
Abortion history***					
	**Yes**	25 (25)	15 (14.85)			
	**No**	75 (75)	86 (85.15)		0.07
Ovarian surgery history****					
	**Yes**	2 (2)	0 (0)		
	**No**	98 (98)	101 (100)		0.49
Infertility history****					
	**Yes**	4 (4)	1 (0.1)		
	**No**	96 (96)	100 (99.9)		0.21
Menstrual status***					
	**Regular**	60 (60)	95 (94)		
	**Irregular**	40 (40)	6 (6)		< 0.001
Menorrhagia***					
	**Yes**	79 (79)	77 (76.2)		
	**No**	21 (21)	24 (23.8)		0.38
*Data presented as Mean ± SD, independent samples *t* test. **Data presented as Mean ± SD, Mann-Whitney's test. ***Data presented as n (%), Chi-squared test. ****Data presented as n (%), Fisher's test. Interquartile range a) 0–2, b) 0–2, c) 0–5.4. BMI: Body mass index, CS: Cesarean section, IUD: Intra-uterine device, NVD: Normal vaginal delivery, OCP: Oral contraceptive pill					

**Table 2:** Hormonal changes and menstrual cycle in the participants in the 1 ${\;}^{\text{st}}$ and 4 ${\;}^{\text{th}}$ months

	**Patient**		**Healthy**				
**Variables**	**1 ${\;}^{\text{st}}$ month**	**4 ${\;}^{\text{th}}$ month**	**1 ${\;}^{\text{st}}$ month**	**4 ${\;}^{\text{th}}$ month**	P-value*	P-value**	**Effect size**
	**Menstrual duration**	6.45 ± 1.98	6.30 ± 1.47	6.65 ± 0.64				6.64 ± 0.65	0.27 ${\;}^{\text{c}}$	0.30 ${\;}^{\text{c}}$	
**P-value**	0.20 ${\;}^{\text{d}}$		0.31 ${\;}^{\text{d}}$				
**Length of menstrual cycle**	28.57 ± 5.97	28.17 ± 4.33	28.02 ± 1.68	28.11 ± 1.37	0.97 ${\;}^{\text{c}}$	0.52 ${\;}^{\text{c}}$	
**P-value**	0.76 ${\;}^{\text{d}}$		0.12 ${\;}^{\text{d}}$				
**Variables**	**1 ${\;}^{\text{st}}$ month**	**4 ${\;}^{\text{th}}$ month**	**1 ${\;}^{\text{st}}$ month**	**4 ${\;}^{\text{th}}$ month**	P-value*	P-value**	**Effect size**
	**TSH (Ulu/ml)**	4.21 ± 1.33	3.77 ± 1.37	3.08 ± 1.14				3.00 ± 1.19	< 0.001^a^	0.13 ${\;}^{\text{c}}$	0.99
**P-value**	< 0.001 ${\;}^{\text{d}}$		0.08^b^				
**TPO (IU/ml)**	35.14 ± 15.49	35.98 ± 15.62	28.99 ± 11.37	32.65 ± 19.75	< 0.001 ${\;}^{\text{c}}$	0.13 ${\;}^{\text{c}}$	0.54
**P-value**	0.95 ${\;}^{\text{d}}$		0.05^b^				
**Prolactin (ng/ml)**	17.14 ± 6.29	17.09 ± 5.69	17.73 ± 4.95	17.84 ± 4.42	0.46^a^	0.69^a^	
**P-value**	0.89^b^		0.06^b^				
**AMH (ng/ml)**	4.52 ± 3.13	1.94 ± 1.34	8.00 ± 2.38	7.93 ± 2.35	< 0.001 ${\;}^{\text{c}}$	< 0.001^a^	2.54
**P-value**	0.02 ${\;}^{\text{d}}$		0.34^b^				
Data presented as Mean ± SD. a) Independent samples *t* test. b) Paired *t* test. c) Mann-Whitney's test. d) Wilcoxon Sum-Rank Test. *In 1 ${\;}^{\text{st}}$ month between groups. **In 4 ${\;}^{\text{th}}$ month between groups. AMH: Anti-Mullerian hormone, TPO: Thyroid per-oxidase, TSH: Thyroid stimulating hormone							

**Table 3:** Multiple regression models for predicting menstrual duration, menstrual length, TSH, TPO, prolactin, and AMH adjusting the confounding variables

	**Unstandardized coefficients**			
**Dependent variable**	**B**	**Std. error**	**t**	**P-value**
	**Menstrual duration^1^**	-0.57			0.17	-0.33	0.74
**Menstrual length^2^**	1.36	0.558	2.44	0.02
**TSH^3^**	1.008	0.554	1.82	0.07
**TPO^4^**	-9.19	5.37	-1.71	0.09
**Prolactin^5^**	1.40	1.90	0.73	0.46
**AMH^6^**	3.22	0.96	3.33	0.002
AMH: Anti-Mullerian hormone, TPO: Thyroid per-oxidase, TSH:Thyroid stimulating hormone. Independent variables include: (1–6) Female age, BMI, age first pregnancy, duration use of contraceptive, sport, moodat				

## 4. Discussion

The current study aimed to assess the menstrual disorder and the AMH, TSH, TPO, and prolactin levels in female patients with COVID-19. No significant difference was observed in the 1 ${\;}^{\text{st}}$ and 4 ${\;}^{\text{th}}$ months regarding menstrual disorders, TPO, and prolactin levels in the patient group. The mean level of AMH in the healthy group was higher than in the patient group at both times. A relationship was observed between TSH and COVID-19 in the 1 ${\;}^{\text{st}}$ month, but no significant relationship was found in the 4 ${\;}^{\text{th}}$ month in this regard.

The results showed that no significant differences were observed between the changes in menstrual cycle duration and length in the 1 ${\;}^{\text{st}}$ and 4 ${\;}^{\text{th}}$ months in the 2 study groups. This finding is consistent with that of Edelman et al. (20), who reported the association between COVID-19 vaccination and a small change in cycle length but not in menses length. On the contrary, Kezhen et al. (21) reported menstrual volume decrease or cycle prolongation in one-fifth of the patients. Menstrual changes can be due to temporary changes in sex hormone levels and suppression of ovarian function, which quickly resume after recovery. There was also an association between the severity of COVID-19 and menstrual irregularities (22). Another study's results showed that the cause of menstrual disorders is negative psychological emotions and that COVID-19 does not aggravate these disorders (23).

A comparison of AMH levels in the healthy and patient groups revealed that the patients had lower AMH levels than the healthy group; this finding is inconsistent with the results of Ding's co-authors (24). Moreover, Yegin et al. (25) found that the mean level of AMH was significantly lower in the COVID-19 group with severe anxiety than in the group with moderate anxiety. Unlikely, Kezhen et al. (21) reported no connection between COVID-19 and AMH level. Ovarian damages such as decreased ovarian reserve and hormonal disorders in the genital tract are involved in reducing AMH levels. BSG and CTSL are both expressed at high levels in cumulus cells, which is the entry point for SARS-CoV-2. As a result, the mature ovum may be at risk of infection and COVID-19 virus transmission. Also, the aldosterone angiotensin renin system (ACE2-mediated) is involved in reproductive processes such as folliculogenesis, steroidogenesis, ovum maturation, and ovulation. Aldosterone angiotensin renin is suppressed by COVID-19, which can cause various disorders, including endothelial dysfunction, inflammation, damage to the reproductive system, and ultimately, reduced levels of AMH (24). It has also been shown that stress and anxiety play a role in lowering AMH levels (25).

Furthermore, the findings of this study demonstrated that the patients' TSH and TPO levels were higher than those of non-patients in the first month; however, no significant differences were observed in TSH and TPO levels between the 2 groups in the 4 ${\;}^{\text{th}}$ month. Study (26) reported that TPO levels increase after COVID-19 disease.

In studies (27, 28) it was found that COVID-19 causes thyroid disorders and changes in thyroid hormone levels that improve over time. Also, a study reported that patients' TSH levels depended on the symptoms such that high TSH levels and hypothyroid symptoms were observed in patients with olfactory decline following the COVID-19 disease (28). Mechanisms that may cause these changes include the direct effect of the virus on the pituitary gland and the indirect effect of the virus in which various systemic changes like the activation of various pro-inflammatory cytokines due to virus infection or its treatment can lead to hormonal changes in the pituitary axis of the endocrine glands.

Another study showed the widespread use of antiplatelet agents, anticoagulants, and corticosteroids in multisystem involvement in COVID-19 infection and endothelial dysfunction, inflammation, vascular and thrombotic events (26). For example, acetylsalicylic acid, by altering plasma protein binding, increases the free hormone fraction, alters the conversion of free thyroxine (fT4) to free tri-iodothyronine (fT3), and finally temporarily suppresses TSH. This eventually leads to a decrease in thyroid hormone production.

In addition, treatment with dexamethasone causes a decrease in endogenous TSH and active fT3 by blocking the conversion processes of plasma protein and deiodinase activity by suppressing the pituitary axis (29). Other studies have shown that heparin increases fT4 and fT3 levels and slightly decreases TSH levels by displacing thyroid hormones from their binding proteins (30, 31).

In the present study, no significant relationship was observed between COVID-19 and prolactin levels in the 1 ${\;}^{\text{st}}$ and 4 ${\;}^{\text{th}}$ months. Unlike the study by Ding et al., who observed that prolactin levels increased in COVID-19 patients (24). Another study found that acute stress increased prolactin levels (32). The difference in the findings can be because stress and anxiety levels in the COVID-19 pandemic vary from person to person and from community to community. As a result, prolactin levels in the COVID-19 pandemic vary in different populations, too.

Our study limitations were: a) low sample size due to the high cost of tests, and b) the hormone levels were determined regardless of the stage of the menstrual cycle just shortly after exposure to SARS-CoV-2. Given that psychological factors are considered an important factor in these disorders, it is suggested that further studies be conducted with larger sample sizes and between different groups in different communities. Also, prolactin level changes require a longer time and inflammatory process, which is recommended to be investigated in studies with a longer period.

## 5. Conclusion

Based on the present research findings, the general prevalence of menstrual and hormonal disorders, especially AMH and TSH levels were associated with COVID-19. It was further revealed that COVID-19 creates a heavy financial burden for the individual and also a great social burden for the country. By adopting appropriate strategies, preventive measures can be taken to reduce the complications of these disorders. Hence, there is a need for further research and taking as well as practical preventive measures so that these disorders do not lead to serious complications in these women in the future.

##  Data Availability

The data sets used and analyzed during the current study are available from the corresponding author upon reasonable request.

##  Author Contributions

Sh. Jahanian Sadatmahalleh and Z. Haghipour contributed to the conception and design of the study; Sh. Jahanian Sadatmahalleh, Z. Haghipour, and F. Razavinia did the literature search; Sh. Jahanian Sadatmahalleh, Z. Haghipour, and M. Nasiri performed the statistical analysis; and Sh. Jahanian Sadatmahalleh, Z. Haghipour, F. Razavinia, and M. Nasiri wrote the first draft of the manuscript. All authors contributed to the manuscript revision, and all read and approved the submitted version.

##  Conflict of Interest

The authors declare that there is no conflict of interest.

‏

## References

[bib1] Kılçar M, Turgut ÜK, Bozkurt KK, Bayhan G (2024). Effects of permanent placental injury due to severe acute respiratory syndrome coronavirus 2 infection during pregnancy on the feto-placental circulation: A cross-sectional study. Rev Assoc Med Bras.

[bib2] Joshi S, Bhatia A, Tayal N, Jha LK, Gupta P (2022). Rare multisystem inflammatory syndrome in young adult after COVID-19 immunization and subsequent SARSCoV-2 infection. J Assoc Physicians India.

[bib3] Sharma I, Kumari P, Sharma A, Saha SC

[bib4] Zhang H, Li H-B, Lyu J-R, Lei X-M, Li W, Wu G, et al (2020). Specific ACE2 expression in small intestinal enterocytes may cause gastrointestinal symptoms and injury after 2019-nCoV infection. Int J Infect Dis.

[bib5] Athar F, Karmani M, Templeman NM (2024). Metabolic hormones are integral regulators of female reproductive health and function. Biosci Rep.

[bib6] Ma L, Xie W, Li D, Shi L, Mao Y, Xiong Y, et al

[bib7] Ding T, Zhang J, Wang T, Cui P, Chen Z, Jiang J, et al

[bib8] Li H, Liu S-M, Yu X-H, Tang S-L, Tang C-K (2020). Coronavirus disease 2019 (COVID-19): Current status and future perspectives. Int J Antimicrob Agents.

[bib9] Honorato‐Sampaio K, Pereira VM, Santos RA, Reis AM (2012). Evidence that angiotensin‐(1-7) is an intermediate of gonadotrophin‐induced oocyte maturation in the rat preovulatory follicle. Exp Physiol.

[bib10] Stanton R, To QG, Khalesi S, Williams SL, Alley SJ, Thwaite TL, et al (2020). Depression, anxiety and stress during COVID-19: Associations with changes in physical activity, sleep, tobacco and alcohol use in Australian adults. Int J Environ Res Public Health.

[bib11] Song S, Choi H, Pang Y, Kim O, Park H-Y (2022). Factors associated with regularity and length of menstrual cycle: Korea nurses’ health study. BMC Women's Health.

[bib12] Anto-Ocrah M, Valachovic T, Chen M, Tiffany K, DeSplinter L, Kaukeinen K, et al (2023). Coronavirus disease 2019 (COVID-19)-related stress and menstrual changes. Obstet Gynecol.

[bib13] Lee W, Mok A, Chung JP (2021). Potential effects of COVID-19 on reproductive systems and fertility; assisted reproductive technology guidelines and considerations: A review. Hong Kong Med J.

[bib14] Liu C, Mu C, Zhang Q, Yang X, Yan H, Jiao H (2021). Effects of infection with SARS-CoV-2 on the male and female reproductive systems: A review. Med Sci Monit.

[bib15] Edlow AG, Li JZ, Ai-ris YC, Atyeo C, James KE, Boatin AA, et al (2020). Assessment of maternal and neonatal SARS-CoV-2 viral load, transplacental antibody transfer, and placental pathology in pregnancies during the COVID-19 pandemic. JAMA Netw Open.

[bib16] Reid PC, Coker A, Coltart R (2000). Assessment of menstrual blood loss using a pictorial chart: A validation study. BJOG.

[bib17] Darney BG, Boniface ER, Van Lamsweerde A, Han L, Matteson KA, Cameron S, et al (2023). Impact of coronavirus disease 2019 (COVID‐19) vaccination on menstrual bleeding quantity: An observational cohort study. BJOG.

[bib18] Kanwal M, Irfani I, Afzal N, Saba E, Dahar A (2023). Awareness, perceptions and future plan for bbilateral tubal ligation as a method for contraception in Pakistani women. J Soc Obstet Gynaecol Pak.

[bib19] Karena ZV, Shah H, Vaghela H, Chauhan K, Desai PK, Chitalwala AR, et al (2022). Clinical utility of mifepristone: Apprising the expanding horizons. Cureus.

[bib20] Edelman A, Boniface ER, Benhar E, Han L, Matteson KA, Favaro C, et al (2022). Association between menstrual cycle length and coronavirus disease 2019 (COVID-19) vaccination: A US cohort. Obstet Gynecol.

[bib21] Li Kezhen, Chen G, Hou H, Liao Q, Chen J, Bai H, et al (2021). Analysis of sex hormones and menstruation in COVID-19 women of child-bearing age. Reprod Biomed Online.

[bib22] Khan SM, Shilen A, Heslin KM, Ishimwe P, Allen AM, Jacobs ET, et al (2022). SARS-CoV-2 infection and subsequent changes in the menstrual cycle among participants in the Arizona CoVHORT study. Am J Obstet Gynecol.

[bib23] Ozimek N, Velez K, Anvari H, Butler L, Goldman KN, Woitowich NC (2022). Impact of stress on menstrual cyclicity during the coronavirus disease 2019 pandemic: A survey study. J Women's Health.

[bib24] Ding T, Wang T, Zhang J, Cui P, Chen Z, Zhou S, et al (2021). Analysis of ovarian injury associated with COVID-19 disease in reproductive-aged women in Wuhan, China: An observational study. Front Med.

[bib25] Yeğin GF, Desdicioğlu R, Seçen Eİ, Aydın S, Bal C, Göka E, et al (2022). Low anti-Mullerian hormone levels are associated with the severity of anxiety experienced by healthcare professionals during the COVID-19 pandemic. Reprod Sci.

[bib26] Yanachkova V, Stankova T, Staynova R (2023). Thyroid dysfunction as a long-term post-COVID-19 complication in mild-to-moderate COVID-19. Biotech Biotechnol Equip.

[bib27] Khoo B, Tan T, Clarke SA, Mills EG, Patel B, Modi M, et al

[bib28] Tsivgoulis G, Fragkou PC, Karofylakis E, Paneta M, Papathanasiou K, Palaiodimou L, et al (2021). Hypothyroidism is associated with prolonged COVID-19-induced anosmia: A case-control study. J Neurol Neurosurg Psychiatry.

[bib29] Mohammed AH, Yousif AM, Jabbar SA, Ismail PA (2021). Assessment of thyroid function in COVID-19 patients. Tabari Biomed Stu Res J.

[bib30] de Noriega Echevarría I, García-Salido A, Muñoz-Calvo MT, Argente J (2017). Elevated thyroid hormone levels following low molecular weight heparin administration. An Pediatr (Barc).

[bib31] Burekovic A, Halilovic D, Sahbaz A (2022). Hypothyroidism and subclinical hypothyroidism as a consequence of COVID-19 infection. Med Arch.

[bib32] Chaudhuri A, Koner S (2018). A study of correlation of perceived stress with serum prolactin levels in newly diagnosed cases of hypothyroidism in females in an Urban population of a developing country. Int J Res Rev.

